# Severe Headache and Acute Blindness: A Case of Pituitary Apoplexy

**DOI:** 10.1002/ccr3.72207

**Published:** 2026-03-05

**Authors:** Abdul‐Rahman Faiza, Adwoa Agyemang Adu‐Gyamfi, Kwaku Asare‐Ankomah, Kwadwo Faka Gyan, Solomon Gyabaah

**Affiliations:** ^1^ Directorate of Internal Medicine, Komfo Anokye Teaching Hospital Kumasi Ghana

**Keywords:** blindness, headache, pituitary adenoma, pituitary apoplexy, visual field

## Abstract

Pituitary apoplexy is a rare, life‐threatening syndrome that commonly occurs in the adenomatous pituitary gland. It presents with a myriad of severe neuroendocrine and ophthalmological signs and symptoms. Early diagnosis and prompt treatment are vital in the management of pituitary apoplexy. This case report describes a 56‐year‐old woman who reported to the neurology clinic with 1 week's history of visual loss, and a severe headache, on the background of a 2‐year history of pituitary adenoma with poor follow‐up. The patient underwent transsphenoidal surgery. She regained vision in the right eye, but no perception of light in the left eye. Patients with headaches and impaired visual field should be screened for pituitary adenoma. Signs and symptoms may be reversible with early diagnosis and prompt treatment of pituitary apoplexy.

## Key Clinical Message

Clinicians should consider pituitary apoplexy a possibility in patients with pituitary adenoma, presenting with acute headaches and visual field defects. If detected early and treated promptly, the signs and symptoms of pituitary apoplexy may be reversible.

**Abbreviations**
ACTH: adrenocorticotropic hormone
CT scan: computed tomography scan
IV fluid: intravenous fluid
Post‐op: post‐operation
T3: triiodothyronine
T4: thyroxine
TSH: thyroid stimulating hormone

## 1 Introduction

Pituitary apoplexy is an uncommon clinical emergency that occurs when there is a hemorrhage or infarction of the pituitary gland [1, 2]. It is a rare, life‐threatening syndrome that occurs in 1.5%–27.7% of individuals with pituitary adenoma [3]. Pituitary apoplexy may cause cerebral infarction through mechanical compression and/or cerebral vasospasm, which commonly involves the internal carotid artery, anterior cerebral artery, and middle cerebral artery [4]. It occurs in both the adenomatous and non‐adenomatous or normal pituitary gland, but commonly in the adenomatous pituitary gland [1]. It is characterized by headache, visual impairment, altered consciousness due to infarction and hemorrhage in the pituitary gland, and hemodynamic instability with neuroendocrine abnormalities. We report the case of a 56‐year‐old woman with pituitary apoplexy who presented with blindness and severe headache.

## 2 Case History/Examination

We report a 56‐year‐old woman who presented to the neurology clinic with complaints of worsening headaches for over 2 years, acute onset of visual loss associated with an episode of projectile vomiting. Upon enquiry, she had been experiencing blurred vision for 3 years and reported to a peripheral facility, where she was given eyeglasses to aid her vision. A year later, she developed headaches that progressively worsened and had a head Computed Tomography (CT) scan done, which was significant for a pituitary adenoma measuring 3 cm by 2 cm. She was referred to the neurosurgery clinic; however, she failed to attend and follow up because she felt frightened by the results. A week prior to presentation at the neurology clinic, she experienced loss of her vision, which prompted further evaluation.

On clinical examination, she was conscious and alert with stable vital signs, a blood pressure of 121/80 mmHg, pulse rate of 80 bpm, temperature of 36.7°C and a respiratory rate of 18 bpm. A confrontation visual field test revealed bitemporal hemianopia. Extraocular muscle movements were normal.

## 3 Differential Diagnosis, Investigations, and Treatment

Her prolactin level was low, measuring 2 ng/L (normal range of 4.79–23.3 pmol/L), free T4 (thyroxine) was low, 7.1 pmol/L (normal range of 12.0–22.0 pmol/L), free T3 (Triiodothyronine) was also low, < 1.7 pmol/L (normal range of 3.1–6.8 pmol/L), and serum cortisol was normal at 669 nmol/L (140–690 nmol/L). However, adrenocorticotropic hormone (ACTH) was low at 1.0 pg/mL (normal range of 2.8–64.6 pg/mL) and glycated hemoglobin was 5.9% (normal < 5.7%).

A CT scan of the head performed from skull base to vertex showed a cystic mass forming a figure of eight with an enhancing rim, measuring 4.6 × 2.7 with no hemorrhage in the Sella turcica. There was compression of the optic chiasma. The visualized paranasal sinuses were clear. The results were consistent with pituitary apoplexy with likely compression of the optic chiasma.

Humphrey visual field test was carried out. No perception of light was observed in the left eye. The reliability indices for the visual field in the right eye were within normal range. It showed right temporal hemianopia. Fundoscopic examination had normal findings.

**FIGURE 1:**
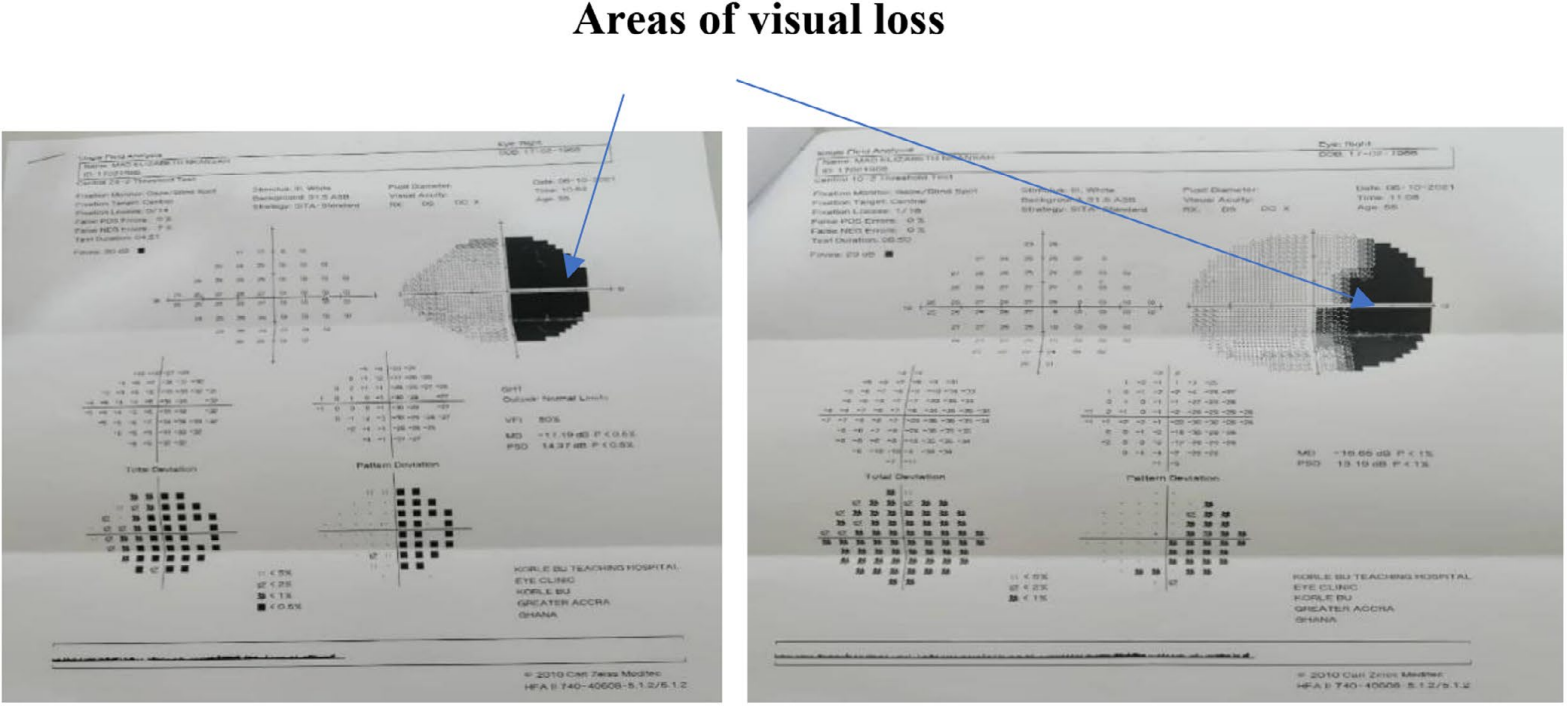
Humphrey visual field test showing right temporal hemianopia (arrows).

**FIGURE 2:**
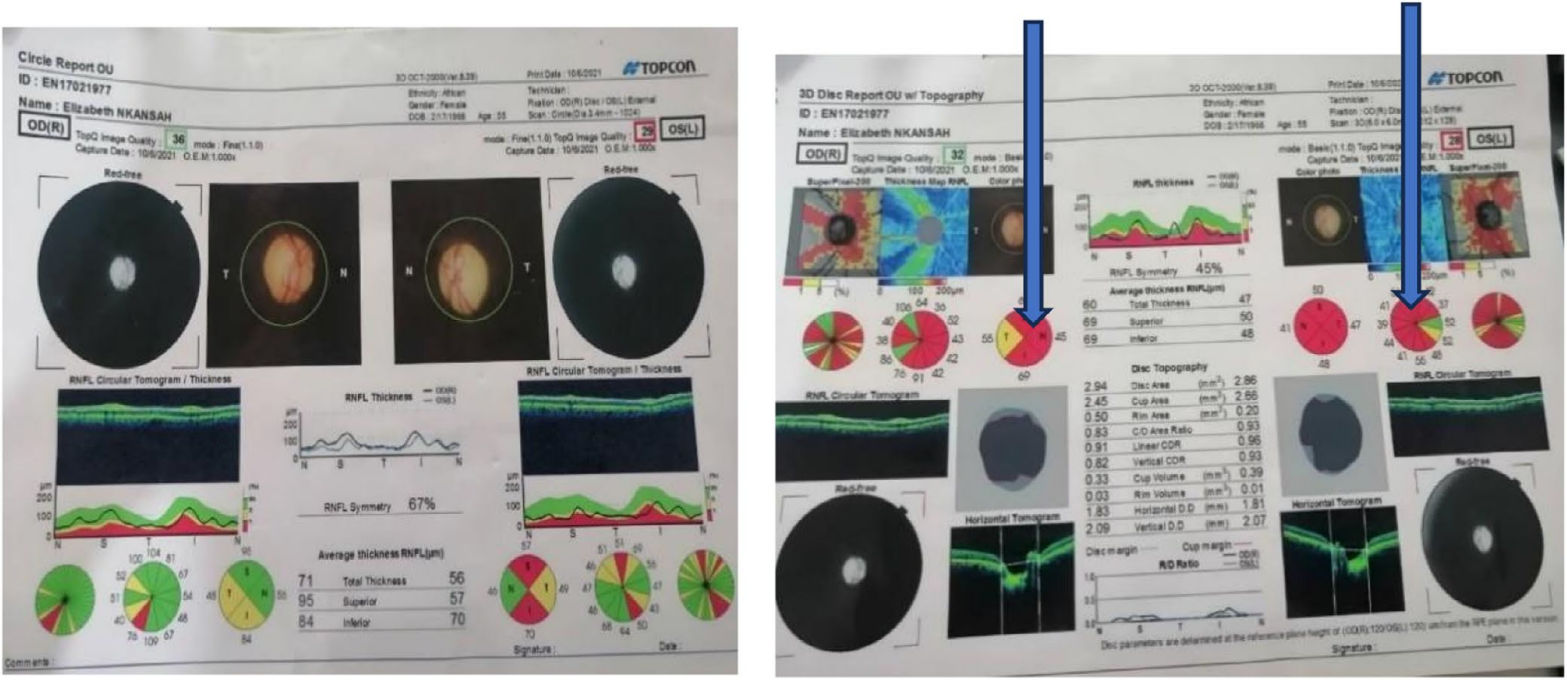
Optical coherence tomography of the optic nerve for both the right and left eye. Picture above shows thinning of the optic nerve in both eyes (OD). Arrow shows the anatomic/structural location of the nerve supply to the opposite location of the fields of vision. Neuro retinal rim has thinned out due to loss of nerve fibers in the inferior, nasal, and superior. This thinning out areas are marked as red.

Optical coherence tomography of the optic nerve corresponded to the visual field tests. Both visual field test and optical coherence tomography are reflective of mass in the sella region (Figures 1 and 2).

The patient was admitted and started on prednisolone 60 mg daily. The headaches subsided, but visual loss persisted. Her blood pressure dropped to < 100 systolic on day three of admission, necessitating the introduction of oral fludrocortisone 0.2 mg daily, after which her blood pressure normalized.

She underwent an endoscopic assisted transsphenoidal microscopic pituitary resection of pituitary adenoma. Operative findings included Coca‐Cola‐like fluid from the central aspect of the tumor and a grayish yellow shellable tumor (fibrous capsule, pruned out sella floor). She recovered after surgery, gained vision in the right eye, evidenced by the ability to count fingers on the 2nd postoperative day, but vision was lost in the left eye. The histopathology report was suggestive of a pituitary adenoma.

## 4 Outcome and Follow‐Up

The patient was doing well on oral hydrocortisone 200 mg daily with no post‐surgical complications. A repeat of her thyroid function test showed TSH 0.500 μU/L, free T4 17.43 pmol, Free T3 = 2.55 pmol/L. Serum ACTH was 1.7 pg/mL, which was still low, and serum cortisol was normal at 609 nmol/L. However, the patient has been lost to follow‐up for a year now.

## 5 Discussion

The case fatality rate of pituitary apoplexy can be significant when it is not diagnosed and treated early. Recent studies report mortality rates ranging from 0% to 15.3% [5]. Up to 20% of cases of pituitary apoplexy are sub‐clinical but have radiological and pathological indications of hemorrhagic infarction [6]. Approximately 80% of patients with pituitary apoplexy may have a deficiency in one or more hormones produced at the anterior pituitary, most commonly ACTH [1]. It affects both visual acuity and visual field due to the involvement of the optic chiasm, and there may also be cranial nerve involvement, especially cranial nerves III, IV, Va, V2, and VI, which pass through the cavernous sinus.

Pituitary apoplexy has a wide and variable spectrum of clinical presentation, but most commonly presents with a sudden onset of severe retro‐orbital headache that may be generalized, visual impairment, and ophthalmoplegia [4, 7]. Other manifestations include nausea, vomiting, photophobia, meningism, neuroendocrine instability, and memory deficit [8]. The frequent headache seen in pituitary apoplexy has been postulated to be due to meningeal irritation, dura mater compression, enlargement of sellar walls, or involvement of the superior division of the trigeminal nerve inside the cavernous sinus [1, 9]. The visual impairment and ophthalmoplegia are due to involvement of the optic nerve, chiasma, or optic tract, as well as the oculomotor nerve, trochlear nerve, and abducens nerve due to their vulnerability at the cavernous sinus [1, 4]. The patient in this report experienced a worsening headache for over 2 years, which later became associated with visual field impairment. Following these symptoms, the patient was diagnosed with a pituitary adenoma with the aid of a head CT scan. However, the patient declined surgery after the pituitary adenoma was detected.

Early diagnosis and prompt treatment are vital in the management of pituitary apoplexy [10]. Unnecessary delays may lead to the irreversibility of some symptoms even after the best surgical intervention [3, 11]. Pituitary apoplexy's endocrine and neuroophthalmological prognosis are inextricably linked to how well the disease is managed in the acute and subacute stages [12]. Indeed, people with severe neurological or ophthalmological impairment can improve dramatically if treated properly, whereas those with modest signs/symptoms can have a poorer fate if the diagnosis and treatment are delayed or they are treated inappropriately. The use of psychotherapy has also been seen to play an important role in managing pituitary disease with severe headaches. Cognitive behavioral therapy reduces fear; this psychological support lowers anxiety, which can amplify symptoms, and improves adherence to medical treatment and readiness for surgical intervention [9].

The patient underwent a successful surgery. Postoperatively, neither diabetes insipidus nor hypopituitarism occurred as evidenced by normal thyroid function tests and normal prolactin levels. She, however, couldn't regain vision in her left eye as well as her right temporal field. It is important to note that delayed surgery can have a negative impact on visual recovery [3].

Patient was lost to follow‐up. Delayed follow‐up in pituitary apoplexy is often related to inadequate patient understanding of the condition's ability to deteriorate rapidly. Failure to communicate the potential risk of permanent visual loss and endocrine complications can decrease adherence to follow‐up [11].

In conclusion, patients with headaches and associated impaired visual field and acuity should be screened for pituitary adenoma. Signs and symptoms may be reversible with early diagnosis and prompt treatment of pituitary apoplexy. Assembling a multidisciplinary team is the best approach in the management of patients with pituitary apoplexy.

## Author Contributions

**Abdul‐Rahman Faiza:** conceptualization, investigation, writing – original draft. **Adwoa Agyemang Adu‐Gyamfi:** writing – original draft, writing – review and editing. **Kwaku Asare‐Ankomah:** writing – review and editing. **Kwadwo Faka Gyan:** writing – original draft, writing – review and editing. **Solomon Gyabaah:** conceptualization, supervision, project administration, writing – original draft, writing – review and editing.

## Funding

The authors have nothing to report.

## Consent

Written informed consent was obtained from the patient for publication.

## Conflicts of Interest

The authors declare no conflicts of interest.

## Data Availability

The data that support the findings of this study are available from the corresponding author upon reasonable request.
